# Spontaneous Gastric Perforation in a Healthy Child Associated With *Sarcina ventriculi* Infection

**DOI:** 10.1155/crpe/6955634

**Published:** 2026-05-04

**Authors:** Dejan Nikolic, Sukrita Mysore, Matthew Boelig, Bilal Khan, Emily Scattergood, Debrah Meislich

**Affiliations:** ^1^ Department of Pathology, Cooper University Health Care, Cooper Medical School of Rowan University, Camden, New Jersey, USA, rowan.edu; ^2^ Department of Pediatric Gastroenterology, University of Maryland, Baltimore, Maryland, USA, umaryland.edu; ^3^ Department of Surgery and Pediatrics, Sidney Kimmel Medical College of Thomas Jefferson University, Philadelphia, Pennsylvania, USA, jefferson.edu; ^4^ Department of Pediatric Radiology, Cooper University Health Care, Cooper Medical School of Rowan University, Camden, New Jersey, USA, rowan.edu; ^5^ Department of Pediatric Infectious Diseases, Cooper University Health Care, Cooper Medical School of Rowan University, Camden, New Jersey, USA, rowan.edu

## Abstract

Spontaneous gastric perforation is a rare but morbid condition in children. Recent reports have suggested that finding *Sarcina ventriculi*, a rare, Gram‐positive anaerobic bacterium in the stomach, could be associated with gastric ulcers and rarely gastric perforation, predominantly in adults. Here, we describe a case of an 11‐year‐old otherwise healthy child who presented with acute onset of severe, diffuse abdominal pain, tenderness, and distension and was found to have peritonitis and shock. The emergent exploratory laparotomy revealed a 4‐cm gastric perforation, and histopathologic examination of the subtotal gastric resection showed abundant growth of *Sarcina ventriculi* at the site of perforation. To our knowledge, this represents one of the very few reported cases and possibly the first well‐documented case occurring in a previously healthy child with no identifiable predisposing conditions.

## 1. Introduction

*Sarcina ventriculi* is an uncommon Gram‐positive anaerobic coccus, whose role in disease is debated, often considered a commensal but occasionally thought to be a pathogen [1–4]. This bacterium is a carbohydrate fermenter capable of surviving and proliferating in acidic environments, including the stomach. *S. ventriculi* is identified by light microscopy, owing to the unique large cell size and their arrangements in cuboid clusters [1, 2]. While it can be detected incidentally in the feces of healthy individuals, particularly those with vegetarian diets, an increasing recognition of *Sarcina* in surgical pathology specimens has raised ongoing debate regarding colonization versus pathogenicity. Reports link *Sarcina* to gastric ulcers and, rarely, perforation, predominantly in adults [1, 2, 4]. Clinical manifestations range from asymptomatic colonization to chronic dyspepsia, abdominal pain, ulcers, emphysematous gastritis, perforation, and even death. Pediatric cases are less common but have been associated with risk factors such as mucosal damage, gastrointestinal dysmotility, or obstruction due to variety of conditions [3, 5–10] as nicely summarized in a recent systematic review of published pediatric reports by Hadjiyannis and colleagues [3]. To date, fewer than 25 pediatric reports exist, and here we present a clinically important and rare pediatric case describing gastric perforation associated with *Sarcina ventriculi* infection in an otherwise healthy child without known predisposing gastrointestinal or neurological conditions.

## 2. Case Report

**FIGURE 1:**
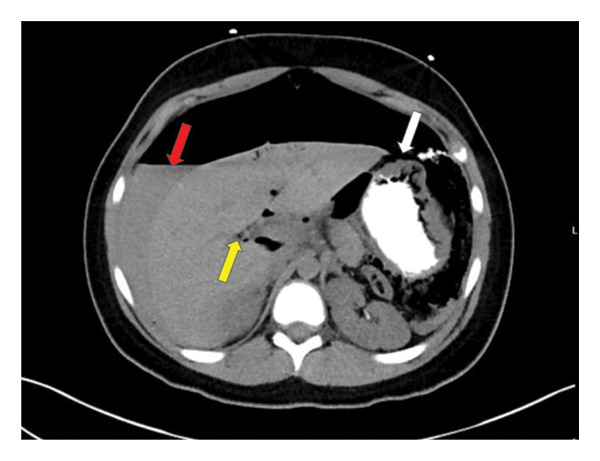
Abdominal computed tomography (CT) scan with PO contrast, axial view, demonstrates discontinuity of the stomach at the level of the fundus along the greater curvature, indicating rupture (white arrow). Large pneumoperitoneum is seen, and a large amount of free fluid containing diluted ingested oral contrast (red arrow) is observed. There is a small amount of peripheral air within the left hepatic lobe, indicating portal venous gas (yellow arrow).

This case follows the course of an 11‐year‐old girl with a remote past medical history of mild intermittent asthma on no treatment, who was visiting New Jersey with her family. The patient was born and raised in the Northeast region of the United States. There was no recent medication use or unusual food exposure prior to presentation. She presented to an outside emergency department with acute onset of severe, diffuse abdominal pain, tenderness, and distension and was quickly transferred to our hospital, where she was found to be in shock and to have peritonitis. Urgent abdominal computed tomography (CT) scan with contrast (Figure 1) demonstrated a large pneumoperitoneum and gastric perforation. Emergent exploratory laparotomy revealed ascites and a 4‐cm necrotic gastric perforation on the greater curvature of the stomach that was managed with stapled wedge resection and omental patch. The CT imaging did not demonstrate, nor did the surgeon observe any evidence of a foreign body. Microscopic examination of hematoxylin‐and‐eosin (H&E)–stained slides of the subtotal gastric resection revealed extensive overgrowth of bacteria with morphological features consistent with the size and tetrad morphology characteristic of *S. ventriculi* at the site of gastric perforation (Figure 2(a)). The uniquely large size of *S*. *ventriculi* cells and arrangements in cuboidal clusters seen in an H&E–stained section are diagnostic (Figure 2(b)), [1–4]. Strikingly, no *S. ventriculi* organisms were present in other areas of the subtotal gastrectomy specimen (data not shown). Gram, Giemsa, Gomori methenamine silver (GMS), and periodic acid–Schiff (PAS) stains were also performed. As expected, the bacteria stained positively with Gram (Supporting Information (available here)), GMS, and Giemsa stains but were PAS‐negative (images not shown). In virtually all cases, light microscopy with H&E and Gram staining is sufficient for the diagnosis, as it was in our case. Molecular confirmation is rarely necessary, and it was not pursued. Postoperatively, the patient was admitted to the critical care unit. She was treated with piperacillin–tazobactam, amoxicillin–sulbactam, and fluconazole. Once *Sarcina* was diagnosed, the proton pump inhibitor pantoprazole was added. The patient was discharged on hospital day 14 on pantoprazole, and at follow‐up 6 months postoperatively, she was doing well with no sequela.

FIGURE 2Gastric perforation site, hematoxylin and eosin (H&E) stain. (a) Low‐power photomicrograph showing extensive basophilic bacterial overgrowth within necrotic gastric tissue, morphologically consistent with Sarcina ventriculi (40×; scale bar = 200 µm). (b) High‐power photomicrograph demonstrating characteristic Sarcina ventriculi morphology, consisting of cuboid‐shaped, basophilic‐stained cocci measuring approximately 2‐3 µm, arranged in tetrads and packets with division in perpendicular planes (400×; scale bar = 20 µm).

**Figure:**
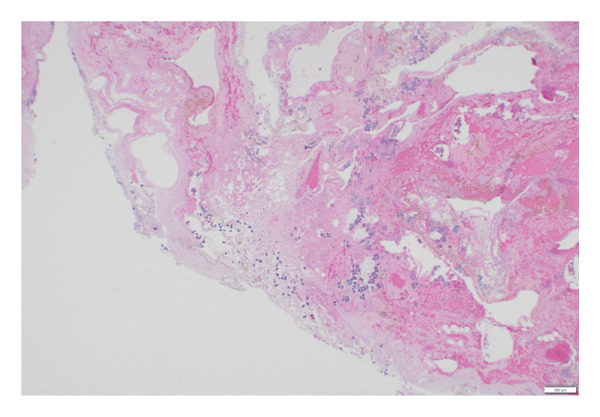
(a)

**Figure:**
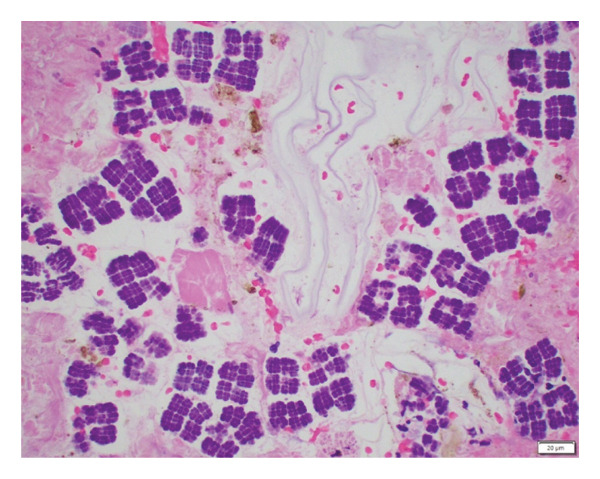
(b)

## 3. Discussion

While frequently detected incidentally in the feces of healthy individuals, an increasing recognition of *Sarcina* in surgical pathology has raised ongoing debate regarding colonization versus pathogenicity [1–4]. The literature is dominated by adult reports linking its presence in the stomach to severe complications including emphysematous gastritis and gastric perforation, mainly in individuals with underlying gastrointestinal disease [1, 2, 4] with only a limited number of reported cases involving children [3, 5–10]. *Sarcina* is well studied in the veterinary literature, as the organism is a gas‐forming fermenter causing lethal gastric bloating–like syndrome in animals. The organism can survive and thrive within extremely low pH environments, including the stomach environment, and utilizes fermentation as a singular energy source, with the subsequent production of ethanol, carbon dioxide, hydrogen, and acetic acid [1, 3]. In humans, pathogenesis remains uncertain, and it is unclear why some colonized individuals remain asymptomatic while others suffer life‐threatening complications [1–4]. It has been hypothesized that *Sarcina* could damage gastrointestinal mucosa through local accumulation of acetaldehyde and ethanol, while carbon dioxide production could cause abdominal distension and emphysematous gastritis seen in some of the patients [1]. A preexisting mucosal defect, such as an ulcer, is thought to provide a nidus for emphysematous gastritis to develop rather than direct invasion of *Sarcina* into the gastric wall [2]. Of the few case reports of *Sarcina* in children, most have been described in those with gastrointestinal or neurological conditions that predispose to mucosal injury and delay gastric emptying favoring the overgrowth of the organism in nutrient‐rich environment and exacerbating mucosal damage [3]. Our patient is unique as she lacked risk factors typically associated with severe *Sarcina* infection. While some patients present with chronic symptoms such as nausea, dyspepsia, or abdominal pain, others, like our patient, develop acute rapidly progressive disease [1–3]. Although *Sarcina* can be an incidental finding on biopsy [1–4], in this case, its presence was confined solely to ulcerated tissues at the perforation site (Figure 2(a)), not elsewhere in the subtotal gastrectomy specimen, suggesting its causal role. If just a colonizer, *Sarcina* probably would occupy different random locations of the stomach instead of being confined only to the perforation site. It is unclear what favored abundant growth of the organism in our patient, perhaps complicating initial mucosal injury resulting in catastrophic complication. No alternative explanation was found, as history, imaging, and histopathology revealed only soft foodstuff, without foreign bodies. On follow‐up, the patient remains well without gastrointestinal complications. To our knowledge, this represents one of the very few reported cases and possibly the first well‐documented case occurring in a previously healthy child with no gastrointestinal or neurological risk factors highlighting the need for additional studies. By reporting this case, we hope to broaden awareness that gastric perforation caused by *S*. *ventriculi* should be on the differential for a child with acute onset abdominal pain and pneumoperitoneum even without a history of delayed gastric emptying or other types of gastric disease.

## Funding

No funding was received for this manuscript.

## Disclosure

The earlier version of this work has been presented as a Poster Abstract at Pediatric Academic Societies Virtual Meeting, according to the following link: https://virtual2021.pas-meeting.org/fsPopup.asp?PresentationID=858429&26mode=presInfo. Session Name: Gastroenterology/Hepatology: e‐Posters, #: EP‐140.888.

## Ethics Statement

Our institution does not require ethical approval for reporting an individual case with no identifiable data included. Accordingly, Institutional Review Board (IRB) review was deemed exempt in accordance with institutional policy for single‐patient case reports.

## Consent

Written informed consent for publication of this case was obtained from the patient’s mother.

## Conflicts of Interest

The authors declare no conflicts of interest.

## Supporting Information

Site of gastric perforation, Gram stain. High‐power photomicrograph showing Gram‐positive coccoid organisms morphologically consistent with Sarcina within necrotic tissue. The organisms measure approximately 3.0 µm in diameter and are arranged in characteristic tetrads and clusters (400×; scale bar = 20 µm).

## Supporting information


**Supporting Information** Additional supporting information can be found online in the Supporting Information section.

## Data Availability

Data sharing is not applicable to this article as no datasets were generated or analyzed during the current study.
