# Supplementary material for: Spontaneous Gastric Perforation in a Healthy Child Associated With Sarcina ventriculi Infection
Source: Case Rep Pediatr. 2026 May 4;2026:6955634. doi: 10.1155/crpe/6955634 (PMC13136845; doi:10.1155/crpe/6955634)
Supplement: Supplementary file 1 — Supporting Information Additional supporting information can be found online in the Supporting Information section. [file CRPE-2026-6955634-s001.docx]

**Supplementary material.**

**As expected, the bacteria stained positively with Gram stain.**


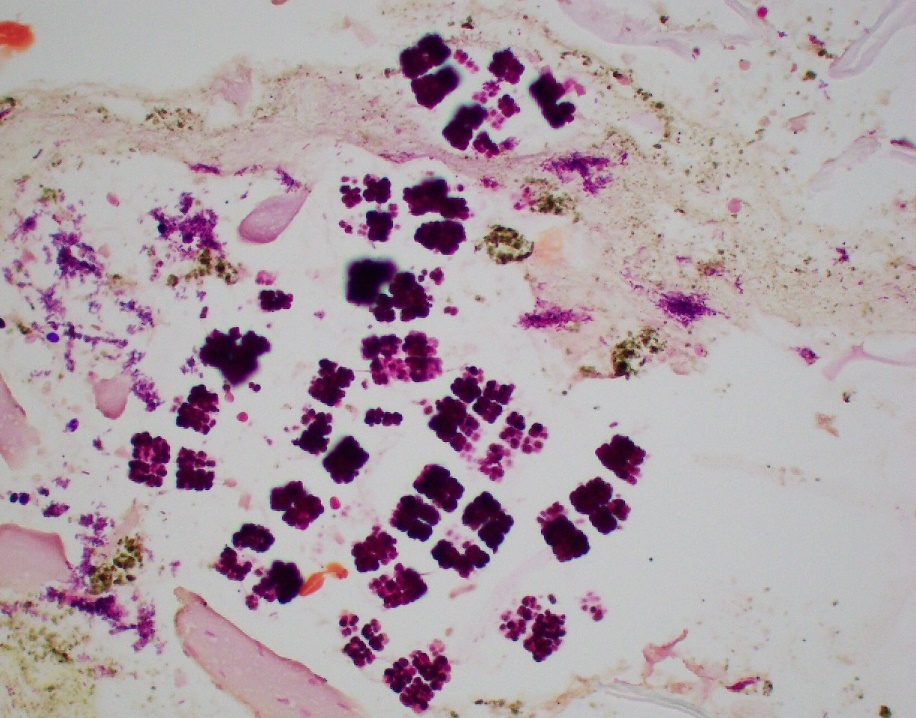


**The same figure containing the scale bar measurement displaying 20 µm. FYI, this scale bar is the burned information in the image that cannot be edited:**


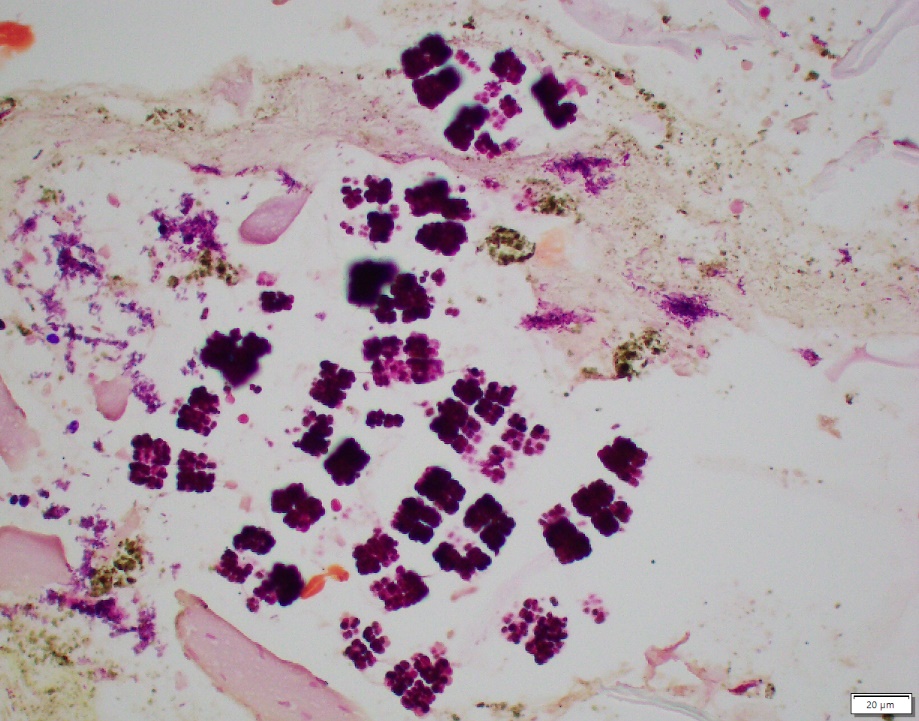


**Please feel free to edit and/or utilize either image.**
